# Raciocínio Clínico em Cardiologia: Passado, Presente e Futuro

**DOI:** 10.36660/abc.20220002

**Published:** 2022-08-24

**Authors:** Evandro Tinoco Mesquita, Mayara Gabriele Toledo, Rodrigo da Silva Garcia Prieto, Amanda Cunha Soares, Eduardo Thadeu de Oliveira Correia

**Affiliations:** 1 Complexo Hospitalar de Niterói Niterói RJ Brasil Complexo Hospitalar de Niterói , Niterói , RJ – Brasil; 2 Universidade Federal Fluminense Hospital Universitário Antônio Pedro Niterói RJ Brasil Universidade Federal Fluminense – Hospital Universitário Antônio Pedro , Niterói , RJ – Brasil; 3 Unigranrio Duque de Caxias RJ Brasil Unigranrio , Duque de Caxias , RJ – Brasil; 4 Universidade Federal Fluminense Pós-Graduação em Ciências Cardiovasculares Niterói RJ Brasil Universidade Federal Fluminense – Pós-Graduação em Ciências Cardiovasculares , Niterói , RJ – Brasil

**Keywords:** Erros de Diagnóstico, Cardiologia, Sistema Cardiovascular, História da Medicina

## Abstract

O raciocínio clínico nasceu 2500 anos atrás com Hipócrates, tendo evoluído ao longo dos séculos, e se tornado uma mistura de arte e ciência. Várias personalidades ao longo da história contribuíram para melhorar a acurácia diagnóstica. Contudo, o erro diagnóstico é ainda comum e causa um grande impacto nos sistemas de saúde. Para lidar com esse desafio, vários modelos de raciocínio clínico surgiram para sistematizar o processo de pensamento clínico. Este artigo descreve a história do raciocínio clínico e os métodos atuais de raciocínio diagnóstico, propõe um novo modelo de raciocínio clínico chamado Raciocínio Integrativo, e traz perspectivas sobre o futuro do raciocínio clínico.

## Introdução

O diagnóstico clínico nasceu há 2500 anos com Hipócrates, tendo evoluído ao longo dos séculos, e se tornado uma mistura de arte e ciência. Muitas personalidades ao longo da história da medicina contribuíram para a melhoria da acurácia diagnóstica. Contudo, o erro diagnóstico ainda é muito comum, com estudos prévios com pacientes em tratamento ambulatorial nos EUA mostrando uma prevalência de 5% de erros. ^1 , 2^ Um relatório da Organização Mundial da Saúde (OMS) mostrou que cerca de 138 milhões de pessoas são afetadas por erros médicos todo ano, dos quais 2,6 milhões perderam suas vidas. ^3^

Sabe-se que a capacidade diagnóstica diferencial e a acurácia diagnóstica tendem a melhorar com a experiência clínica. ^4^ Estudos recentes, no entanto, mostram que é difícil para os médicos tomarem decisões assertivas. Esse fenômeno é agravado pelo fato de que o raciocínio clínico e os processos cognitivos envolvidos na tomada de decisão pelo médico não são bem abordados nos currículos das faculdades de medicina. ^4^ Médicos mais experientes normalmente necessitarão de menos dados para alcançar a mesma conclusão que profissionais menos experientes ou em treinamento. ^4^

Ensinar raciocínio clínico é desafiador, pelo fato de ser subjetivo e requerer múltiplas habilidades (p.ex., avaliação da história do paciente, exame físico, solicitação de exames complementares adequados, e análise de diagnósticos diferenciais). ^5^ Em cardiologia, o desafio é ainda maior, dada a sobreposição de sintomas de diferentes doenças cardiovasculares. ^5^ Recentemente, muitos autores sugeriram ferramentas para sistematizar o processo do pensamento clínico, reduzir erros diagnósticos, e facilitar o ensino a estudantes de graduação e médicos menos experientes. ^6^ Contudo, revisões que abordam de maneira abrangente o raciocínio clinico em cardiologia são escassas. Assim, este artigo descreve a história do raciocínio clínico e os métodos atuais de raciocínio diagnóstico, e propõe um novo modelo de raciocínio clínico chamado Raciocínio Integrativo, e traz perspectivas sobre o futuro do raciocínio clínico.

### Passado

O raciocínio clínico é o processo mental utilizado pelos médicos para gerar hipóteses diagnósticas para uma doença. Tem um papel importante na capacidade médica em formular e testar hipóteses diagnósticas, resolver problemas, e tomar decisões assertivas. ^7^ Assim, o raciocínio clínico é considerado o centro da competência médica e uma parte integral da prática clínica, combinado com experiências acumuladas ao longo da carreira. ^8^ Portanto, o raciocínio clínico é um processo contínuo, não linear, extremamente complexo que requer processos cognitivos, aquisição de conhecimentos teóricos e práticos, capacidade de resolução de problemas, e metacognição. ^9^

Historicamente, o diagnóstico clínico surgiu com Hipócrates (há 2380 anos, em 370 a.c.). Muitos médicos deram importantes contribuições ao longo da história, com a descoberta de doenças e seus processos fisiopatológicos, e desenvolvimento de tecnologias para aprimorar o exame físico. ^10^ Particularmente para as doenças cardíacas, os desenhos de Andreas Vesalius e a descrição da circulação sanguínea e da fisiologia cardíaca por William Harvey foram os primeiros passos para a fundação da cardiologia moderna. ^11 - 14^ Anos depois, Giovanni Battista publicou seu grande trabalho: “ *De Sedibus et Causis Morborum per Anatomen Indagatis* ” (“Dos sítios e causas de doenças, por autópsia”), pelo qual foi marcado como fundador da anatomia patológica, o que permitiu a associação de autópsias com o estado clínico do paciente. ^15 , 16^

A escola francesa foi outro grande contribuidor para o nascimento da cardiologia moderna. Corvisart deu sua contribuição importante com a valorização de uma anamnese à beira do leito e exame físico detalhados. ^17^ Além disso, Corvisart reintroduziu e aperfeiçoou o método de percussão torácica no diagnóstico clínico, traduzindo o manuscrito de Leopold von Auenbrugger “Inventum Novum” do latim para o francês. ^17^ Tal fato levou ao seu reconhecimento como o fundador da cardiologia clínica. Ainda da escola francesa, René Théophile Hyacinthe Laennec inventou o estetoscópio, instrumento indispensável para se realizar um exame físico completo, capaz de trazer pontos chave ao processo cognitivo de formulação do diagnóstico. ^18^ Laennec inventou e descreveu vários termos utilizados até hoje, tais como “murmúrio vesicular”, “som bronquial”, “crepitações”, “ronco”, “pectoriloquia”, “atrito pleural”. ^19^ Outro membro da escola francesa, Marie-François-Xavier Bichat, descobriu a independência do coração em relação ao cérebro, o primeiro passo para se compreender o sistema de condução cardíaca. ^20 , 21^

Passando para a escola germânica, Rudolf Ludwig Karl Virchow e William Osler devem ser reconhecidos por sua importância na construção do pensamento médico no diagnóstico de doenças cardiovasculares. Virchow cunhou termos que são usados ainda hoje, como trombose, embolia, agenesia, cromatina, parênquima, mielina, leucocitose, leucemia, endarterite, amiloide, degeneração, e osteoide, além de descrever o mecanismo de formação de trombos nos vasos sanguíneos, conhecido como tríade de Virchow. ^22 , 23^ Osler destacou a importância da relação médico-paciente, da observação, e do rigor científico, e de avaliar o paciente e seus sintomas em detalhes, descrevendo cada alteração no exame físico. ^24^ Outra grande contribuição de seu trabalho foi a criança da residência médica, em defesa da educação médica continuada. ^25^ Osler também criou o a “Regra de Osler”, em que cada paciente deve receber apenas um diagnóstico que explique sua doença. Essa regra foi seguida até o século vinte, quando os pacientes apresentavam uma baixa expectativa de vida e por isso morriam antes de desenvolverem várias comorbidades. ^25^

Outro importante médico foi o Sir Arthur Ignatius Conan Doyle, da escola de Edimburgo. Sir Arthur Doyle, inspirado pela arte da dedução de seu professor Dr. Joseph Bell, criou o personagem Sherlock Holmes, descrito como um investigador meticuloso, o que mostra como Sir Doyle via a importância da construção de scripts na formulação do diagnóstico. ^26 , 27^

Após o período de estudos anatomopatológicos e análise meticulosa dos sintomas dos pacientes, novas ferramentas foram desenvolvidas para ajudar no diagnóstico em cardiologia, especialmente a eletrocardiografia (1902), pelo fisiologista holandês Willem Einthoven. Então, Dr. Paul Dudley White contribuiu para a descoberta de importantes achados eletrocardiográficos que ainda compõem as listas de problema hoje, com ênfase na descrição da síndrome de Wolff-Parkinson-White. ^28 , 29^

**Figura 1:**
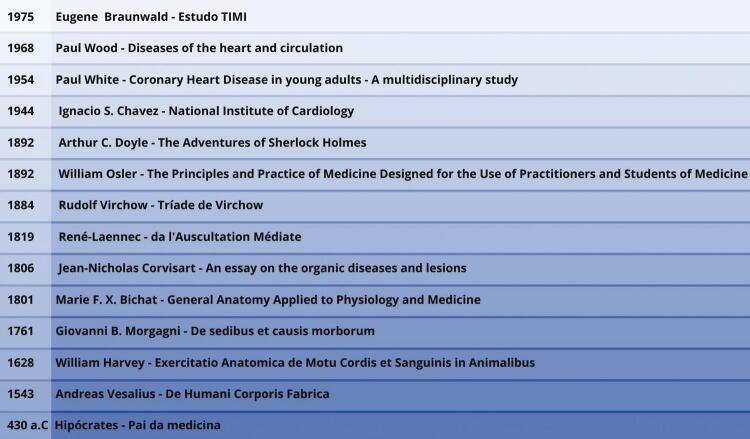
A maioria desses médicos descreveram suas descobertas em seus livros, listados na figura. 17

Outro importante nome na história do desenvolvimento do raciocínio clínico, Paul Hamilton Wood, é reconhecido como um ícone na transição da cardiologia antiga para a cardiologia moderna, assim como Paul Dudley White e Ignacio Chávez Sánchez. ^30 , 31^ Wood redefiniu a síndrome de Eisenmenger como um estado patológico atribuído à hipertensão pulmonar com um *shunt* bidirecional ou invertido, o que minimiza seus efeitos. Wood também descreveu que a hipertensão pulmonar é capaz de produzir vasoconstrição arterial pulmonar, ^30^ a qual pode ser revertida por injeção de acetilcolina na artéria pulmonar, e propôs que vasoconstrição arterial pulmonar agiria como um mecanismo protetor contra o edema pulmonar agudo. ^31 , 32^ Ignacio Chávez Sánchez contribuiu para a descrição dos achados clínicos da hipertensão pulmonar. Além de trazer a cardiologia mexicana à linha de frente, construiu as bases para a inclusão do humanismo como a principal força motriz por trás das ações médicas, o que é importante para o estabelecimento de uma boa relação médico-paciente, e favorece a coleta de informações na anamnese e no exame físico. ^33 , 34^ Finalmente, o cardiologista Eugene Braunwald, que desenvolveu um método de cálculo, que posteriormente se tornou conhecido como fração de ejeção, essencial na avaliação da condição de insuficiência cardíaca. ^35^ Em 1967, Eugene Braunwald e seu grupo identificaram os principais determinantes do consumo de oxigênio: o desenvolvimento de tensão, e a velocidade e frequência de contração. ^36^ Em 1984, ele criou o grupo de estudo TIMI ( *Thrombolysis in Myocardial Infarction* ), ^37^ que envolveu vários hospitais e comparou estreptoquinase, um antigo medicamento, com alteplase (TPA), demonstrando a superioridade do segundo, o que foi importante para a sua aprovação pelo *Food and Drug Administration* dos EUA. ^36^ Contribuições importantes para a fundação do raciocínio clínico em cardiologia estão descritas na Tabela 1 e Figura 1 .

**Tabela 1:** Séculos de trabalho para o entendimento do diagnóstico em cardiologia, descritos por médicos e suas principais contribuições

Médicos	Contribuições
Hipócrates	Pioneiro na documentação e interpretação do estudo de caso ^10^
Andreas Vesalius	A correlação anatomoclínica está eternizada no atlas de anatomia "De Humani Corporis Fabrica" ^11^
William Harvey	Descrição do coração como uma bomba e do sistema circulatório como um circuito fechado ^14^
Giovanni Morgagni	Definição de estenose mitral, angina pectoris, endocardite na obra "De Sedibus et Causis Morborum per Anatomen Indagatis" ^15^
Jean-Nicolas Corvisart-Desmarets	Publicou o primeiro tratado em cardiologia e reintroduziu o método de Auenbrugger de percussão torácica para o diagnóstico clínico ^17,19^
René Theopphile Laennec	Inventou o estetoscópio, descreveu conceitos de semiologia respiratória (De L'auscultation médiate) e sons cardíacos ^17^
Marie François Xavier Bichat	Descoberta do automatismo cardíaco ^20^
Rudolf Virchow	Descrição do mecanismo de formação de trombo, conhecido como “Tríade de Virchow”) ^23^
William Osler	Criou regra de Osler, fundou a residência médica, e participou da descoberta das plaquetas ^24,25^
Arthur Conan Doyle	Descreveu alterações vasomotoras da neurossífilis e defendeu a busca exaustiva por dados clínicos para o diagnóstico e, baseado nisso, criou o personagem Sherlock Holmes. ^27^
Paul Dudley White	Participou da descoberta da Síndrome Wolff-Parkinson-White e da criação da *American Heart Association* , e defendeu a relação entre estilo de vida e doença arterial coronariana ^.29^
Ignácio Chávez Sánchez	Fundou o Instituto Nacional de Cardiologia no México e escreveu artigos sobre síncope, hipertensão essencial, hipertensão pulmonar, e doença cardíaca isquêmica associada a aterosclerose. ^33,34^
Paul Hamilton Wood	Trabalhou com cardiopatia congênita, doença valvar reumática, e hipertensão pulmonar, e escreveu o livro "Diseases of the heart and circulation". ^30^
Eugene Braunwald	Descreveu a relação entre níveis de lipoproteína de baixa densidade (LDL) e risco de ataque cardíaco; participou no grupo de estudo TIMI, que levou à aprovação da alteplase pela FDA para o tratamento de síndrome coronariana aguda. ^37^

FDA: Food and Drug Administration.

### Presente

Hoje, o raciocínio clínico tem sido estudado por pesquisadores de várias áreas, tais como medicina, educação, psicologia clínica, e psicologia cognitiva. ^38 - 40^ Essas áreas investigam o processo de diferentes maneiras, mas são unânimes em destacar que é necessário habilidade para a tomada de decisão médica. ^39^ Nesse contexto, estudos em psicologia cognitiva sobre cognição trouxeram *insights* importantes aos processos mentais. ^40^ Essa área investiga questões tais como metacognição, memória, reconhecimento de padrão, percepção, atenção, criatividade, raciocínio, e resolução de problemas. ^41 , 42^ Nesse sentido, podemos afirmar que a psicologia cognitiva reúne a cognição estrutural com processos de raciocínio crítico, tais como raciocínio dedutivo e indutivo, o que gera o chamado pensamento sistêmico.

O raciocínio clínico é governado por dois sistemas de pensamento, conhecido como “sistema 1”, ou raciocínio clínico geral, e “sistema 2”, ou raciocínio clínico particular; a interação entre esses dois sistemas determina o pensamento. Tais sistemas baseiam-se no livro de Daniel Kahneman, “Rápido e devagar duas formas de pensar”. ^43^ O sistema 1 é rápido, automático, impulsivo, e intuitivo, e geralmente atua sem controle voluntário. Por outro lado, o sistema 2, é calculista, deliberado, e analítico, e responsável pelo raciocínio e tomada de decisão. Esse último sistema busca focar no objeto de interesse e evitar distrações para alcançar um objetivo. ^43^

Durante o processo de raciocínio clínico, o reconhecimento de padrão utiliza o sistema 1. Médicos experientes, após anos de prática e estudos de casos, tendem a formular o diagnóstico final principalmente pelo sistema 1, uma vez que o longo caminho de acúmulo de conhecimento os permitiu acumular uma série de padrões, conhecidos como scripts de doenças. O método usado por esses médicos mais experientes é também o que a Psicologia Cognitiva chama de “heurística”, que consiste em simplificar a busca de soluções de um problema, para minimizar o esforço da mente. ^44^ Por outro lado, estudantes e médicos que iniciaram recentemente suas carreiras tendem a usar o sistema 2, uma vez que o acúmulo de experiência e de conhecimento ocorre de forma gradual.

A heurística é um modelo de raciocínio rápido, não analítico e intuitivo para se estabelecer, de maneira não consciente, a relação entre a apresentação do paciente e padrões de doença armazenados na memória de longo prazo. A heurística é caracterizada pela rapidez com que o médico levanta hipóteses diagnósticas. ^44 , 45^ Essa atividade é acionada no modelo tradicional de raciocínio científico chamado método hipotético-dedutivo, que consiste em encontrar uma solução a um problema utilizando tentativas (conjeturas, hipóteses, teorias), e eliminando erros. ^46^ Esse método nasceu na epistemologia científica, o resultado de discussões sobre método indutivo *versus* método dedutivo. ^47^ De acordo com esse pensamento, o médico busca por uma solução para um problema (doença) por meio de possíveis respostas, em um processo de tentativas, conjunturas, e refutação. ^47^ O conjunto de dados obtidos sobre um problema do paciente é registrado, e inclui a primeira impressão médica, a história, e o exame físico do paciente. ^48^ As hipóteses encontradas são divididas em hipóteses principais e alternativas. O médico testa as hipóteses até encontrar um grau de probabilidade que será usado para confirmar o diagnóstico e excluir outros, e guiar o plano terapêutico individualizado. ^37^ Nesse modelo de raciocínio, a experiência do médico em compreender as doenças é colocado como um determinante da probabilidade de um diagnóstico correto. Nesse sentido, pesquisadores começaram a questionar como podemos compreender o padrão de raciocínio usado pelo médico experiente e apresentá-lo ao médico iniciante. ^49^ A pesquisa nessa área começou a se intensificar nos anos 70, quando estudos sobre o raciocínio clínico mostraram que o que diferenciava estudantes dos médicos experientes não era o modelo cognitivo, e sim a assertividade e a qualidade das hipóteses. Assim, com base nesse ponto de vista, a acurácia da hipótese inicial prediz a acurácia do diagnóstico. ^50^

Os scripts de doença são dados armazenados na memória do médico, acessados mediante apresentação do paciente, bem no início do processo de raciocínio clínico. ^51^ Os scripts são construídos de acordo com a experiência vivida pelos médicos e estudantes, ou seja, são organizados com base nos padrões de doenças analisadas ao longo de suas trajetórias. Quanto maior a frequência com que esses padrões são vistos e discutidos, mais refinados se tornam os scripts. Contudo, a formação e o estabelecimento de scripts de doença por um profissional se dão não somente com sua experiência prática, mas também com estudos e conhecimento teórico. ^51 , 52^

Após aprender e acumular muitos scripts, ao ser apresentado a uma doença de um paciente, o médico acessa diagnósticos memorizados e escolhe a doença que rapidamente vem à mente devido a suas características muito similares às encontradas naquela ocasião. ^44^ Esse processo muito rápido e intuitivo envolve o chamado “viés cognitivo”, caracterizado pelo uso de atalhos que levam a uma direção para simplificar o pensamento, *i.e* ., um viés é uma tendência ou uma distorção a favor ou contra algo. ^45^ Isso pode levar a um diagnóstico incorreto, e consequente transmissão de informação inapropriada ao paciente, e início de terapia inadequada. Existem vários tipos de vieses e, na Tabela 2 , descrevemos os cinco principais tipos na prática clínica, e como reduzir seu impacto sobre o raciocínio diagnóstico. O conhecimento desses vieses pode levar a estratégias analíticas para corrigi-los, reduzindo, provavelmente a ocorrência de erros diagnósticos. ^38 , 53^

**Tabela 2:** Principais vieses na prática clínica e como reduzir seu impacto sobre o raciocínio clínico

Vieses cognitivos	Descrição
Encerramento prematuro	Parar de considerar diagnósticos diferenciais após atingir o diagnóstico inicial. ^46^ É o tipo mais comum de viés no erro diagnóstico segundo o artigo "Diagnostic error in internal medicine" ^63,64^
Disponibilidade	Quando um diagnóstico é definido com a hipótese mais fácil de ser lembrada, sem se pensar muito no caso. ^63,64^
Confirmação	Maior apreciação dos fatos que confirmam o diagnóstico que daqueles que o refutam. ^63,65^
Moldura/enquadramento	A maneira como os dados são apresentados ao médico tem influência no raciocínio e pode levar a erro. ^63,65^
Ancoragem	O diagnóstico mais provável é aquele justificado pela história clínica do paciente. O ponto inicial do raciocínio clínico passa a ser a comorbidade do paciente, reduzindo as possiblidades de outros diagnósticos. ^63,64^

De maneira similar, o chamado “ruído” também pode contribuir para a ocorrência de erros diagnósticos. Esse conceito foi abordado no livro “ *Noise: a flaw in human judgment* ”, também de Daniel Kahenman, que define o conceito como a “variabilidade nos julgamentos que deveriam ser idênticos”. ^40^ Ainda, o livro apresenta dois tipos principais de ruídos, o ruído ocasional, quando fatores externos influenciam as decisões de um indivíduo ou um grupo. E o ruído sistêmico, que descreve a variabilidade indesejada que ocorre quando um grupo de experts tentam avaliar, separadamente, eventos similares. Ter opiniões diferentes é saudável e importante para a medicina e construção do conhecimento. No entanto, quando há variabilidade nos julgamentos que deveriam ser idênticos, o processo de raciocínio torna-se confuso e ainda mais propenso a erro. ^40^ Assim, de acordo com Kahneman, quando uma combinação de vieses e ruídos ocorre, erros complexos ocorrem. ^40^

### Raciocínio Integrativo

**Figura 2:**
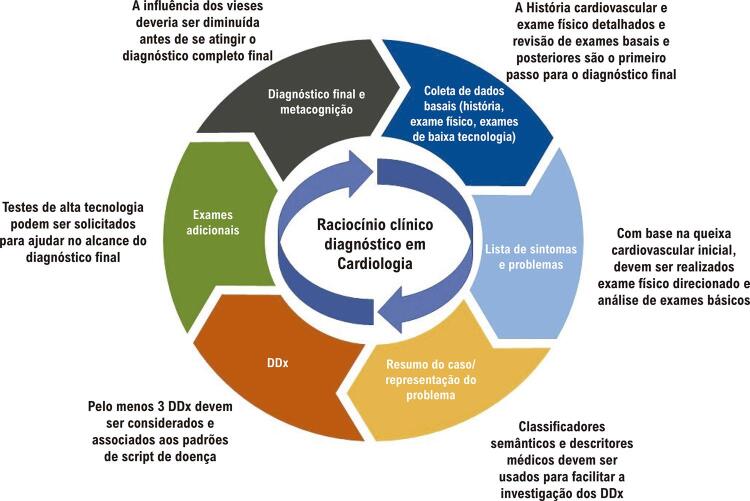
Fluxograma do raciocínio clínico diagnóstico em cardiologia; DDx: diagnósticos diferenciais. 62

Com base em evidências de teorias cognitivas, modelos contemporâneos de diagnóstico clínico e análise dos erros mencionados neste artigo, estruturamos uma proposta para abordar o raciocínio clínico que nós chamamos Raciocínio Integrativo, que engloba os passos detalhados na Figura 2 . O primeiro contato com o paciente consiste em uma história clínica e um exame físico detalhados. Em seguida, o médico deve organizar os dados mais importantes, formulando um quadro de sintomas e uma lista de problemas. Essa etapa é essencial para transformar as queixas trazidas pelos pacientes em qualificadores semânticos e um resumo de casos. Após analisar essa última etapa, passamos para a formulação de hipóteses baseada no conhecimento prévio e padrões aprendidos, e já pensamos em possíveis diagnósticos diferenciais, considerando a epidemiologia. No mínimo, três diagnósticos diferenciais devem ser listados. Se necessário, exames complementares são solicitados, sendo que podem ser necessários testes de custo baixo, médio ou alto. É importante enfatizar que, no modelo proposto, quando as hipóteses diagnósticas são feitas de maneira assertiva, os exames solicitados serão apenas os estritamente necessários. Após essas etapas, o diagnóstico final é alcançado, mas o processo de metacognição deve ser realizado. Quando necessário, o médico pode retornar à coleta de dados da história do paciente, gerar novas hipóteses, e/ou solicitar novos exames. Dessa forma, o médico pode adicionar ou remover novos dados da lista de problemas e gerar novas hipóteses e diagnósticos diferenciais. Retornar aos processos já aplicados envolve metacognição, a qual é definida como a capacidade humana em monitorar e autorregular processos cognitivos, e é baseada na característica humana de estar consciente de suas ações e pensamentos. A metacognição é importante não só para a formulação do diagnóstico final, como também para médicos e estudantes reconhecerem suas limitações, e principalmente para perderem o medo de pedir uma segunda opinião. Esse processo é essencial para o diagnóstico de doenças, uma vez que o pensamento recorrente durante o raciocínio clínico pode conter vieses, ruído, e levar ao erro diagnóstico. ^40 , 45^ A falha no diagnóstico ocorre quando um diagnóstico correto para um problema de saúde não é dado em tempo apropriado (o que pode ser fatal) ou quando esse fato não é explicado ao paciente. ^54^ Outro fator que contribui para se evitar erros diagnósticos é o estímulo de se aprender os processos mentais do raciocínio clínico precocemente durante o treinamento médico. Para isso, desde o início do curso de medicina, os estudantes devem ser encorajados a estabelecerem uma boa relação médico-paciente, juntamente com uma coleta de dados detalhada, envolvimento do paciente e dos familiares no diagnóstico, e revisão cuidadosa dos resultados dos exames. ^54 , 55^ Obter uma história médica completa é de extrema importância para o raciocínio clínico. Em seguida, discussões sobre os casos devem ser realizadas, e o processo proposto acima seguido. Deve-se enfatizar a importância de se formular diagnósticos diferenciais, e revisar dados coletados e as hipóteses propostas. Além disso, os estudantes devem ser encorajados a praticar pedir ajuda a outros profissionais para discutirem o caso, analisarem exames complementares, e formulares o diagnóstico final. ^41^ Ainda, a discussão sobre vieses de ruído e erros diagnósticos deve ser constantemente promovida durante o treinamento. A falha em qualquer ponto do pensamento mental durante o raciocínio clínico pode gerar erros diagnósticos. ^56 , 57^ Além disso, com o avanço de tecnologias, o uso de aplicativos e websites que ajudam na formulação de diagnósticos diferenciais e hipóteses é inevitável e positivo. No entanto, algumas faculdades de medicina, principalmente as mais tradicionais, ainda apresentam certa recusa em estimular tais ferramentas, o que consiste em uma barreira que deve ser superada na melhoria do processo mental do raciocínio clínico tanto do estudante como do médico. ^50 , 57^

### Futuro

Muitas ferramentas são promissoras em melhorar a acurácia diagnóstica e auxiliar médicos a chegarem a um diagnóstico final. Por exemplo, a inteligência artificial e o *Big Data* certamente irão exercer um papel na seleção de doenças com alta probabilidade em cada apresentação de caso. Este é o caso do Isabel Healthcare, ^58^ uma ferramenta médica que ajuda médicos a chegarem a diagnósticos diferenciais, e o estudo CHAMPION que demonstrou a eficácia clínica do sistema de monitoramento hemodinâmico para melhorar o manejo clínico dos pacientes com insuficiência cardíaca sintomática. Esses aparelhos variam desde simples braceletes ou relógios que medem saturação de oxigênio, pressão arterial e frequência cardíaca, até aparelhos hemodinâmicos invasivos que registram o status de volume em pacientes com insuficiência cardíaca. ^59^ Os aparelhos de telemedicina também auxiliarão a coletar dados e direcionar a tomada de decisão à distância. Por fim, a impressão 3D poderá um dia ser usada para orientar cirurgiões cardíacos no planejamento de cirurgias com precisão, evitando desfechos indesejáveis. ^60 , 61^

## Conclusões

O desenvolvimento do raciocínio clínico iniciou-se séculos atrás, e ainda está em progresso constante. No entanto, esse tema não é muito explorado em faculdades e residências médicas. Conforme apresentado neste artigo, o modelo de raciocínio integrativo serve como um modelo em etapas para o raciocínio diagnóstico e remoção de ruídos e vieses, servindo tanto para médicos experientes como para estudantes em treinamento. Estudos futuros são necessários para validar esse modelo.
