# Supplementary material for: Raciocínio Clínico em Cardiologia: Passado, Presente e Futuro
Source: Arq Bras Cardiol. 2022 Aug 24;119(5):793–800. [Article in Portuguese] doi: 10.36660/abc.20220002 (PMC9750203; doi:10.36660/abc.20220002)
Supplement: Translation

# Translation: Clinical Reasoning in Cardiology: Past, Present and Future

**Authors:** Evandro Tinoco Mesquita, Mayara Gabriele Toledo, Rodrigo da Silva Garcia Prieto, Amanda Cunha Soares, Eduardo Thadeu de Oliveira Correia

**Affiliations:** 1 Brazil Complexo Hospitalar de Niterói, Niterói, RJ – Brazil; 2 Brazil Universidade Federal Fluminense – Hospital Universitário Antônio Pedro, Niterói, RJ – Brazil; 3 Brazil Unigranrio, Duque de Caxias, RJ – Brazil; 4 Brazil Universidade Federal Fluminense – Pós-Graduação em Ciências Cardiovasculares, Niterói, RJ – Brazil

**Keywords:** Diagnostic Errors, Cardiology, Cardiovascular System, History of Medicine

## Abstract

Clinical reasoning was born 2,500 years ago with Hippocrates, having evolved over the centuries, becoming a mixture of art and science. Several personalities throughout history have contributed to improving diagnostic accuracy. Nonetheless, diagnostic error is still common and causes a severe impact on healthcare systems. To face this challenge, several clinical reasoning models have emerged to systematize the clinical thinking process. This paper describes the history of clinical reasoning and current diagnostic reasoning methods, proposes a new clinical reasoning model, called Integrative Reasoning, and brings perspectives about the future of clinical reasoning.

## Introduction

Clinical diagnosis was born 2,500 years ago with Hippocrates, having evolved over the centuries, and become a mixture of art and science. Many personalities throughout the history of medicine have contributed to improving diagnostic accuracy. However, diagnostic error is still very common, with previous studies in outpatient clinics in the USA showing a 5% prevalence of errors.^1 , 2^ Furthermore, a report from the World Health Organization (WHO) showed that about 138 million people are affected by medical errors annually, of which 2.6 million have lost their lives.^3^ It is known that the differential diagnostic capacity and diagnostic accuracy tend to improve with clinical experience.^4^ Recent studies however have shown that it is difficult for physicians to make assertive decisions. This phenomenon is further aggravated by the fact that clinical reasoning and cognitive processes involved in the physician’s decision-making process are not well covered in medical schools’ curricula.^4^ Typically, more experienced doctors will need less data to reach the same conclusion as less experienced doctors or doctors in training.^4^

Teaching clinical reasoning is challenging, since it is subjective and requires multiple skills (e.g. history taking, physical examination, to order appropriate complement tests, and to think about differential diagnoses).^5^ In cardiology, the challenge is even greater, due to the overlap of symptoms between different cardiovascular diseases.^5^ Recently, many authors have suggested tools to systematize the clinical thinking process, reduce diagnostic errors and facilitate teaching to undergraduate students and less experienced physicians.^6^ Nonetheless, reviews that address clinical reasoning in cardiology in a comprehensive manner are scarce. Therefore, this paper describes the history of clinical reasoning and current diagnostic reasoning methods, and proposes a new clinical reasoning model, called Integrative Reasoning, and brings perspectives about the future of clinical reasoning.

### Past

Clinical reasoning is the mental process used by physicians to generate diagnostic hypotheses for a disease. It plays an important role in the medical ability to formulate and test diagnostic hypothesis, solve problems, and make assertive decisions.^7^ Thus, it is considered the center of medical competence and an integral part of clinical practice, coupled with the experiences accumulated throughout one’s career.^8^ Therefore, clinical reasoning is a continuous, non-linear, extremely complex process that requires cognitive processes, acquisition of theoretical and practical knowledge, problem-solving capacity, and metacognition.^9^

Historically, clinical diagnosis has emerged with Hippocrates (2,380 years ago, in 370 B.C.). Many physicians have made significant contributions over the history, with the discovery of diseases and their pathophysiological processes, and development of technologies to improve physical examination.^10^ Particularly for heart disease, the drawings of Andreas Vesalius and the description of blood circulation and cardiac physiology by William Harvey were the first steps towards the foundation of modern cardiology.^11 - 14^ Years later, Giovanni Battista Morgagni published his great work: “ *De Sedibus et Causis Morborum per Anatomen Indagatis* ” (Of the sites and causes of disease, investigated by autopsy **)** , for which he was marked as the founder of pathological anatomy, which allowed the association of autopsies with patient’s clinical status.^15 , 16^

The French school was another great contributor to the birth of modern cardiology. Importantly, Corvisart with the valorization of a detailed bedside anamnesis and physical examination.^17^ Beyond that, Corvisart reintroduced and perfected the method of chest percussion in clinical diagnosis, translating Leopold von Auenbrugger’s manuscript “Inventum Novum” from Latin into French.^17^ This fact led to his recognition as the founder of clinical cardiology. Still from the French school, René Théophile Hyacinthe Laennec invented the stethoscope, an indispensable instrument to perform a thorough physical examination, capable of bringing key points for the cognitive process of formulating the diagnosis.^18^ Laennec coined and described several terms used until today, such as “vesicular murmur”, “bronchial sounds”, “crackling”, “snoring”, “pectoriloquy”, “egophony” and “pleural friction rub”.^19^ Another member of the French School, Marie-François-Xavier Bichat discovered the independence of the heart from the brain, the first step to understand the cardiac conduction system.^20 , 21^

Moving on to the German school, Rudolf Ludwig Karl Virchow and William Osler must be acknowledged for their prominence in building medical thinking in the diagnosis of cardiovascular disease. Virchow coined terms that are still used today, such as thrombosis, embolism, agenesis, chromatin, parenchyma, myelin, leukocytosis, leukemia, endarteritis, amyloid, degeneration, and osteoid, in addition to performing the description of the mechanism of thrombus formation in blood vessels, known as Virchow’s triad.^22 , 23^ Osler highlighted the importance of the physician-patient relationship, observation, and scientific rigor, and of assessing the patient and symptoms in detail, describing each change in the physical examination.^24^ Another major contribution of his work was the creation of medical residency, in defense of continuous medical education.^25^ Osler also created the so-called “Osler’s Rule” - each patient should be given only one diagnosis that explains his disease - which was followed until the 20th century when patients had a low life expectancy and therefore died before they developed multiple comorbidities.^25^

Another physician of importance was Sir Arthur Ignatius Conan Doyle, of the Edinburgh school. Sir Arthur Doyle, inspired by the art of deduction of his teacher Dr. Joseph Bell, created the character Sherlock Holmes, described as a meticulous investigator, which shows how Sir Doyle viewed the importance of constructing scripts in the formulation of diagnosis.^26 , 27^

After the period of anatomopathological studies and meticulous analysis of patients’ symptoms, new tools to aid diagnosis in cardiology were developed, especially the electrocardiogram (1902), by the Dutch physiologist Willem Einthoven. Then, Dr. Paul Dudley White contributed to the discovery of important electrocardiographic findings that are still part of problem lists today, with emphasis on the description of the Wolff-Parkinson-White Syndrome.^28 , 29^

Another important name in the history of the development of clinical reasoning, Paul Hamilton Wood, is recognized as an icon in the transition from ancient to modern cardiology, as are Paul Dudley White and Ignacio Chávez Sánchez.^30 , 31^ Wood redefined the Eisenmenger Complex as a pathological state attributed to pulmonary hypertension with an inverted or bidirectional shunt, which would minimize its effects. He also described that pulmonary hypertension is capable of producing pulmonary arterial vasoconstriction,^30^ which can be reversed by injection of acetylcholine into the pulmonary artery, and drafted that pulmonary arterial vasoconstriction would act as a protective mechanism against acute pulmonary edema.^31 , 32^ Ignacio Chávez Sánchez contributed to the description of the clinical findings of pulmonary hypertension. In addition to bringing Mexican cardiology to the forefront, he laid the foundation to include humanism as the great driving force behind medical actions; this is important for the establishment of a good doctor-patient relationship and favors the collection of information in the anamnesis and physical examination.^33 , 34^ Finally, the cardiologist Eugene Braunwald, who developed a calculation method that later became known as the ejection fraction, which became essential to evaluate the condition of heart failure.^35^ In 1967 Eugene Braunwald and his study group identified the main determinants of oxygen consumption: the development of tension, and the velocity and frequency of contraction.^36^ In 1984, he created the TIMI (Thrombolysis in Myocardial Infarction) Study Group,^37^ which involved several hospitals and compared streptokinase, an old drug, with alteplase, demonstrating the superiority of the latter, which was important for the approval of alteplase by the US Food and Drug Administration.^36^ Important contributions to the foundation of clinical reasoning in cardiology are described in Table 1 and Figure 1 .

**Table 1:** Centuries of work for the understanding of diagnosis in cardiology, described by physicians and their main contributions

Doctors	Contributions
Hippocrates	Pioneer in case study documentation and interpretation^10^
Andreas Vesalius	Anatomical-clinical correlation is eternalized in the anatomy atlas "De Humani Corporis Fabrica".^11^
William Harvey	Description of the heart as a pump and of the circulatory system as a closed circuit^14^
Giovanni Morgagni	Definition of mitral stenosis, angina pectoris, endocarditis in the work "De Sedibus et Causis Morborum per Anatomen Indagatis"^15^
Jean-Nicolas Corvisart-Desmarets	Published the first modern treatise on cardiology and reintroduced Auenbrugger's method of chest percussion for clinical diagnosis.^17,19^
René Theopphile Laennec	Invented the stethoscope, described concepts of respiratory semiology (De L'auscultation médiate) and heart sounds^17^
Marie François Xavier Bichat	Discovery of cardiac automatism^20^
Rudolf Virchow	Description of the thrombus formation mechanism, known as the "Virchow Triad"^23^
William Osler	Created the Osler's rule, founded the medical residency, and participated in the discovery of platelets.^24,25^
Arthur Conan Doyle	Described vasomotor changes in Tabes Dorsalis and advocated the exhaustive search for clinical data leading to diagnosis, and based on this, created the character, Sherlock Holmes.^27^
Paul Dudley White	Participated in the discovery of Wolff-Parkinson-White Syndrome and the creation of the American Heart Association, as well as advocated the relationship between lifestyle and coronary artery disease.^29^
Ignácio Chávez Sánchez	Founded the Mexican National Cardiology Institute and authored papers on syncope, essential hypertension, pulmonary hypertension, and ischemic heart disease associated with atherosclerosis.^33,34^
Paul Hamilton Wood	Worked with congenital heart diseases, rheumatic valve disease, and pulmonary hypertension, and wrote the book "Diseases of the heart and circulation".^30^
Eugene Braunwald	Described the relationship between circulating LDL level and risk of heart attack; participated in the TIMI study group that led to FDA approval of alteplase for treatment of acute coronary syndrome.^37^

TIMI: Thrombolysis in Myocardial Infarction; FDA: U.S. Food and Drug Administration.

**Figure 1:** Most of these doctors have left their discoveries described in their books, which are mentioned in the figure. 17

### Present

Today, clinical reasoning has been studied by researchers from various fields, such as medicine, education, clinical psychology, and cognitive psychology.^38 - 40^ These fields investigate the process in different ways, but are unanimous in noting that it is necessary skill for medical decision-making.^39^ In this context, cognitive psychology studies on cognition have brought important insights into mental processes.^40^ This area investigates issues such as metacognition, memory, pattern recognition, perception, attention, creativity, reasoning, and problem-solving.^41 , 42^ In this sense, we can state that cognitive psychology unites structural cognition with critical reasoning processes such as deductive and inductive reasoning, which generates the so-called systems thinking.

Clinical reasoning is governed by two systems of thought, known as “system 1” or general clinical reasoning, and “system 2” or clinical reasoning in particular; the interaction between these two systems determines the thinking. These systems are based on Daniel Kahneman’s book, “Thinking, Fast and Slow”.^43^ System 1 is fast, automatic, impulsive, and intuitive, and often acts without voluntary control. System 2, on the other hand, is calculating, deliberate, and analytical, and is responsible for reasoning and decision making. This last system seeks to focus on the object of interest and avoid distractions to achieve a goal.^43^

During the clinical reasoning process, pattern recognition resorts to system 1. Experienced physicians, after years of practice and case studies, tend to formulate the final diagnosis mainly through system 1, since the long journey of accumulating knowledge has allowed them to store a series of patterns, known as disease scripts. The method used by these more experienced doctors is also what Cognitive Psychology calls “heuristics”, which consists in simplifying the search for solutions when faced with a problem to save the mind effort.^44^ On the other hand, students and doctors who have just started their careers tend to use mainly system 2, since accumulation of experience and knowledge is gradual.

Heuristics is a model of rapid, non-analytical, intuitive reasoning to unconsciously establish the relationship between patient presentation and disease patterns stored in long-term memory. Heuristics are characterized by the rapidity with which the physician raises diagnostic hypotheses.^44 , 45^ This activity is required in the traditional model of scientific reasoning called hypothetico-deductive reasoning, which consists of finding a solution to a problem using attempts (conjectures, hypotheses, theories) and eliminating errors.^46^ This method was born in scientific epistemology, the result of discussions about inductive versus deductive methods.^47^ According to this thinking, the doctor looks for a solution for a problem (disease) through possible answers, in a process of attempts, conjectures, and refutations.^47^ The set of data obtained about the patient’s problem is recorded and includes the first medical impression, the history, and the physical examination of the patient.^48^ The hypotheses found for the case are divided into main and alternative hypotheses. The physician tests the hypotheses until finding a degree of probability that will be used to confirm the diagnosis and exclude others and guide the individualized therapeutic plan.^37^ In this model of reasoning, the physician’s experience in understanding diseases is placed as a determinant of the probability of a correct diagnosis. In this sense, researchers have begun to question how we can understand the reasoning pattern used by the experienced physician and bring it to the novice physician.^49^ The intensification of research in this area began in the 1970s when studies involving clinical reasoning showed that what differentiated students from experienced physicians was not the cognitive model, but the assertiveness and quality of the hypotheses. Therefore, according to this view, the accuracy of the initial hypothesis predicts the accuracy of the diagnosis.^50^

Illness scripts are the data stored in the memory, accessed when the physician is faced with a picture presented by a patient at the very beginning of the clinical reasoning process.^51^ The scripts are formed according to the experience lived by doctors and students, that is, they are organized based on the patterns of diseases analyzed along their trajectory. The more often the patterns are seen and discussed, the more refined the scripts become. However, the formation and establishment of illness scripts by the practitioner occur not only with the practical experience of but with studies and theoretical knowledge.^51 , 52^

After learning and accumulating many scripts, when presented to a patient’s disease, the physician uses of memorized diagnoses and picks the disease that quickly comes to mind because of its very similar characteristics with those seen at the occasion.^44^ This quick and very intuitive process involves the so-called “cognitive bias”, which is characterized by the use of shortcuts that lead in one direction to simplify thinking, *i.e* . a bias is a tendency or a distortion in favor or against something.^45^ This may lead to an incorrect diagnosis, and consequent transmission of inappropriate information to the patient, and initiation of inadequate therapy. There are several types of biases, and in Table 2 we describe the five most important ones in clinical practice and how to reduce their impact on diagnostic reasoning. Awareness of these biases can evoke analytic strategies to correct it, probably reducing the occurrence of diagnostic errors.^38 , 56^

**Table 2:** The most important biases in clinical practice and how to reduce their impact on diagnostic reasoning

Cognitive biases	Description
Premature closure	Stopping to think about differential diagnoses after reaching an initial diagnosis.^46^It is the most common type of bias in diagnostic error according to the article "Diagnostic error in internal medicine"^63,64^
Availability	When the diagnosis is established by the easiest hypothesis to remember, with little thought devoted to the case.^63,64^
Confirmation	Greater appreciation of facts that confirm the diagnostic hypothesis than those that refute it.^63,65^
Framing	The way data is presented to the doctor influences reasoning and can lead to error.^63,65^
Anchoring	The most likely diagnosis is the one that is justified by the patient's history of disease. The starting point for clinical reasoning becomes the patient's comorbidity, reducing the possibilities of other diagnoses.^63,64^

Similarly, the so-called “noise” is also likely to contribute to the occurrence of diagnostic errors. This concept was addressed in the book “Noise: a flaw in human judgment”, also by Daniel Kahneman, who defines this concept as “variability in judgments that should be identical.^40^ Furthermore, the book brings two main types of noise, the occasion noise, when external factors influence the decisions of an individual or a group, and the systemic noise, which describes the unwanted variability that happens when a group of experts tries to separately evaluate similar events. Having different opinions is healthy and important for medicine and knowledge building. However, when there is variability in judgments that should be identical, the diagnostic reasoning process becomes fuzzy and even more error-prone.^40^ Thus, according to Kahneman, when a combination of biases and noise occurs, complex errors occur.^40^

### Integrative Reasoning

Based on evidence from cognitive theories, contemporary models of clinical diagnosis, and analysis of the errors mentioned in this article, we structured a proposal to approach clinical reasoning that we named Integrative Reasoning, which encompasses the steps detailed in Figure 2 . The first contact with the patient consists of a detailed history and physical examination. Next, the physician must organize the most important data, formulating a symptom chart and a problem list. This step is essential to transform the complaints brought by the patients into semantic qualifiers and a case summary. After analyzing this last step, we proceed to the formulation of hypotheses based on previous knowledge and learned patterns, and we already think about possible differential diagnoses, considering the epidemiology. At least three differential diagnoses should be listed. If necessary, complementary exams are requested, and low, medium or high-cost tests may be required. It is important to emphasize that, in the proposed model, when the diagnostic hypotheses are assertively made, the tests ordered will be those that are strictly necessary. After these steps, the final diagnosis is reached, but the process of metacognition must be carried out. When necessary, the physician can return to collection of data from patient’s history, generate new hypotheses, and/or request new tests. In this way, the physician can add or remove new data from the problem list and generate new hypotheses and differential diagnoses. Returning to the processes already applied involves metacognition, which is defined as the human ability to monitor and self-regulate cognitive processes and is based on the human characteristic of being aware of one’s actions and thoughts. Metacognition is important not only for the formulation of the final diagnosis, but also for doctors and students to recognize their limitations and difficulties, and especially to lose the fear of asking for a second opinion. This process is essential for the diagnosis of diseases, because the recurrent thinking during clinical reasoning may contain biases, noise, and lead to diagnostic error.^40 , 45^ Misdiagnosis is defined as the failure to establish a correct diagnosis at the appropriate time for a health problem (which may be life-threatening) or to communicate this explanation to the patient.^54^ Another factor that contributes to avoiding diagnostic errors is the encouragement of learning the mental processes of clinical reasoning early on during medical training. To this end, from the beginning of medical school, students should be encouraged to establish a good doctor-patient relationship in conjunction with detailed data collection, involvement of patient and family in the diagnosis, and careful review of test results.^54 , 55^ A complete medical history is of paramount importance for clinical reasoning. Then, discussions of the cases should be carried out and the process proposed above be followed. It is worth emphasizing the importance of formulating differential diagnoses, and reviewing the data collected and the proposed hypotheses. Also, students should be encouraged to practice asking for help from other professionals to discuss the case, understand the complementary exams, and formulate the final diagnosis.^41^ Moreover, the discussion of noise biases and diagnostic errors should be constantly promoted during training, and failure at any point of mental thinking during clinical reasoning can generate diagnostic errors.^56 , 57^ Furthermore, with the advance of technologies, the use of applications and websites that help in the formulation of differential diagnoses and hypotheses is inevitable and positive. However, some medical schools, especially the more traditional ones, still present a refusal to encourage these tools, and this is a barrier that must be overcome to improve the mental process of clinical reasoning of both students and physicians.^50 , 57^

**Figure 2:** Flowchart of medical reasoning in cardiology. CV: cardiovascular; Hx: history; PE: physical examination; DDx: differential diagnoses. 65

### Future

Many tools hold promise on improving diagnostic accuracy and helping physicians to reach a final diagnosis. For example, artificial intelligence and Big Data will certainly play a role in selecting diseases with higher likelihood in each case presentation. This is the case of Isabel Healthcare,^58^ a medical tool that helps doctors to come up differential diagnoses and the CHAMPION study that demonstrated the clinical effectiveness of the CardioMEMS hemodynamic monitoring system to improve clinical management of patients with symptomatic heart failure. These devices can range from simple bracelets and watches to measure oxygen saturation, blood pressure, and heartbeat, to invasive hemodynamic devices to register volume status in heart failure patients.^59^ Telemedicine devices will also help to collect data and guide decision making from distance. Finally, 3D printing may one day be used to guide cardiac surgeons to plan surgeries with precision, avoiding undesirable outcomes.^60 , 61^

## Conclusions

The development of clinical reasoning began centuries ago and is still in constant progression. However, this subject is not well explored by medical schools and residencies. As presented in this paper, the integrative reasoning model may serve as a simple and stepwise framework for diagnostic reasoning and removal of noise and biases, serving both experienced physicians and students in training. However, future studies are needed to validate this model.
